# Ecchordosis physaliphora: Case report and brief review of the literature

**DOI:** 10.1016/j.radcr.2021.09.049

**Published:** 2021-10-17

**Authors:** Dhairya A Lakhani, Daniel Martin

**Affiliations:** Department of Radiology, West Virginia University, 1 Medical Center Drive, Morgantown, WV 26506

**Keywords:** Chordoma, Ecchordosis Physaliphora, Notochord

## Abstract

Ecchordosis physaliphora is a rare congenital benign hamartomatous lesion originating from nodal cord remnants. This is histopathologically indistinguishable from chordoma, and hence imaging plays a key role in diagnosis. These lesions are hypointense on T1-weighted and hyperintense on T2-weighted images, and follow CSF signal. In contrast to chordoma, Ecchordosis Physaliphora does not demonstrate contrast enhancement. Here, we present a case of 32–year-old male with no prior medical history, who presented to an outside facility for chronic headache workup and incidentally detected indeterminate lytic defect in the bony clivus with a well demarcated smoothly corticated margin. Further assessment with MRI brain showed findings characteristic of Ecchordosis physaliphora, a benign congenital hamartomatous lesion originating from nodal cord remnants requiring no additional follow-up imaging or intervention.

## Background

Ecchordosis physaliphora is a rare congenital benign hamartomatous lesion, found in approximately 2% of autopsies [1,2]. It originates from nodal cord remnants [1]. Typically, this lesion is incidentally detected and is asymptomatic [3]. It occasionally causes mass effect with compression of brainstem or cranial nerves [1,2,4]. It is most commonly found in the retroclival prepontine region of the middle cranial fossa, but can be found anywhere along the midline from the skull base to the sacrum [2,3].

Ecchordosis physaliphora is an imaging diagnosis, as it is histopathologically indistinguishable from chordoma [3]. CT is not a sensitive modality for detection of these lesions, due to posterior fossa artifacts and CSF density of the lesion. On CT it presents as bony clival defect, which is well demarcated and smoothly corticated without any aggressive features. Occasionally, it demonstrates a pathognomonic osseous stalk at its base [1,3]. On MRI, the lesion is hypointense on T1-weighted and hyperintense on T2-weighted images, and follows CSF signal characteristics on T2-Fluid-attenuated inversion recovery (T2-FLAIR) images. In contrast to chordoma, Ecchordosis Physaliphora does not demonstrate contrast enhancement [1,3].

Here we present a case of Ecchordosis physaliphora with classic imaging characteristics along with differential considerations for midline middle cranial fossa masses, to include neoplastic and non-neoplastic lesions.

## Case report

**Fig. 1:**
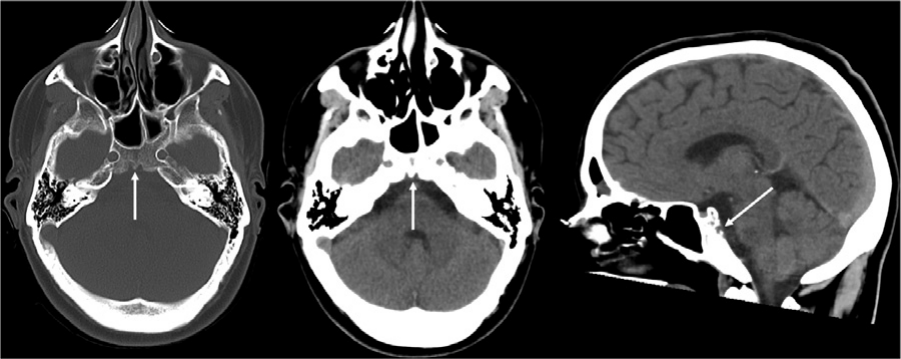
CT Brain without contrast showed an indeterminate lytic defect in the bony clivus with a well demarcated smoothly corticated margin (arrow). This was reported as concerning for malignancy, with recommendation for further evaluation with MRI brain and referral to our tertiary care center for further workup.

The patient is a 32 –year-old male with no prior medical history, who presented to an outside facility for chronic headache workup. On presentation, he was afebrile and had stable vital signs. Initial laboratory workup is unremarkable. CT head without intravenous contrast was performed (Fig. 1), and showed an indeterminate lytic defect in the bony clivus with a well demarcated smoothly corticated margin. This was reported as concerning for malignancy, with recommendation for further evaluation with MRI brain and referral to our tertiary care center for further workup.

**Fig. 2:**
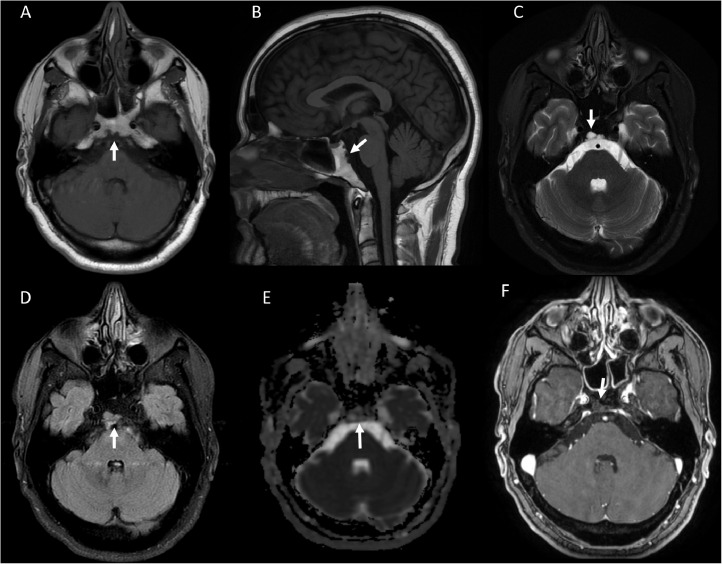
MRI brain with and without contrast was performed at our facility. This demonstrated a well-defined midline retroclival 5 mm x 12 mm cystic lesion (arrow) with hypointense signal on T1-weighted (A, B) and hyperintense signal on T2-weighted images (C), and followed CSF signal on T2-Fluid-attenuated inversion recovery (T2-FLAIR) images (D). Further, this lesion did not restrict diffusion (E) and did not demonstrate abnormal enhancement (F). Based on the midline location and characteristic imaging appearance, the findings were reported as compatible with Ecchordosis physaliphora, a benign congenital hamartomatous lesion originating from nodal cord remnants requiring no additional follow-up imaging or intervention

MRI brain with and without contrast was performed at our facility (Fig. 2). This demonstrated a well-defined midline retroclival 5 mm x 12 mm cystic lesion with hypointense signal on T1-weighted and hyperintense signal on T2-weighted images. The lesion followed CSF signal on T2-Fluid-attenuated inversion recovery (T2-FLAIR) images. Further, this lesion did not restrict diffusion and did not demonstrate abnormal enhancement. Based on the midline location and characteristic imaging appearance, the findings were reported as compatible with Ecchordosis physaliphora, a benign congenital hamartomatous lesion originating from nodal cord remnants requiring no additional follow-up imaging or intervention.

## Discussion

German pathologist Hubert von Luschka first described the finding of pathologic ectopic notochordal tissue at the posterior clivus in 1856 [5]. Existing literature reports some controversy in nomenclature of this entity, and refers to intradural chordoma and ecchordosis physaliphora as actually representing the same entity. Wolfe et al. proposed the term 'intradural chordoma' for all intradural lesions originating from notochordal remnant [6], whereas Rodriguez et al. proposed that all intradural lesions of notochordal remnant origin should be referred as ecchordosis physaliphora until pathologically proven to be chordoma. Currently, these entities are considered distinct pathologies with a shared origin [7].

Neoplastic lesions in the central skull base include metastasis, myeloma and lymphoma originating from the clivus; invasive pituitary macroadenoma; meningioma, metastatic disease, diffuse leptomeningeal glioneuronal tumor and lymphoma originating from the pachymeninges; chordoma and Ecchordosis physaliphora originating from notochord remnants; nasopharyngeal carcinoma (direct invasion from nasopharynx) and chondrosarcoma (originating from the petroclival synchondrosis, off-midline). Additionally, nonneoplastic primary osteofibrous lesions in this region include fibrous dysplasia, ossifying fibroma, Paget's disease and Langerhans' cell histiocytosis [[8], [9], [10], [11]].

The central skull base also houses some anatomic variants, which also do not require any intervention or imaging follow-up. These include craniopharyngeal canal, canalis basilaris medianus, fossa navicularis magna, arrested sphenoid pneumatization, asymmetric pneumatization petrous apex, aberrant internal carotid artery, fibrous dysplasia and cephalocele [[8], [9], [10]].

Ecchordosis physaliphora can be distinguished from its pathologic counterpart chordoma based on its imaging presentation, including the presence of CSF attenuation and the absence of contrast enhancement or osseous destruction. In contrast, chordoma typically presents as a lobulated extra-axial hyperdense soft tissue mass with osseous destruction, with or without internal calcification and mass effect on the brainstem (resulting in characteristic thumb sign). Additionally, chordoma demonstrates heterogenous increased T2 signal, and shows a variable enhancement pattern. [6,7]

## Patient consent

No patient identifiers are disclosed in the current report.
